# A hypervariable intron of the *STAYGREEN* locus provides excellent discrimination among *Pisum fulvum* accessions and reveals evidence for a relatively recent hybridization event with *Pisum sativum*

**DOI:** 10.3389/fpls.2023.1233280

**Published:** 2023-08-25

**Authors:** N. F. Weeden, M. Lavin, S. Abbo, C. J. Coyne, K. McPhee

**Affiliations:** ^1^ Department of Plant Sciences & Plant Pathology, College of Agriculture, Montana State University, Bozeman, MT, United States; ^2^ The Robert H. Smith Faculty of Agriculture, Food and Environment, and the Jacob & Rachel Liss Chair in Agronomy at the Hebrew University of Jerusalem, Rehovot, Israel; ^3^ Plant Germplasm Introduction and Testing Research, Agricultural Research Service (USDA), Pullman, WA, United States

**Keywords:** repetitive DNA, genetic diversity, gene phylogeny, interspecific hybridization, intron variability

## Abstract

An analysis of 82 non-synonymous *Pisum fulvum* accessions for sequence variation in a fragment of the *STAYGREEN* (*SGR*) locus revealed 57 alleles, most of which differed in indel structure. Eight additional *P. fulvum* accessions, each supposedly synonymous with a different accession of the initial group, were also analyzed. In every case the paired synonymous accessions possessed the same SGR sequence but varied slightly for a 6-trait morphological phenotype, indicating that SGR sequence is a much more reliable indicator of accession identity than is a morphological characterization. SGR sequence analysis confirmed our previous finding that *P. fulvum* accessions separate into two allele groups. This division was not supported by results of previous studies that were based on sequences distributed across the entire genome, suggesting that the division may have been produced by selection at a nearby locus and that the SGR phylogeny may not be good indicator of overall relationships within the species. One *P. fulvum* accession, PI 595941 (=JI1796), displayed an SGR sequence outside the variation typical of the species. Instead, its allele resembled alleles limited to a set of *Pisum sativum* landraces from the Middle East, suggesting hybridization between ancestors of PI 595941 and some primitive form of domesticated *P. sativum*. With one exception from the extreme northwest corner of Israel, *P. fulvum* accessions collected north of latitude 35.5° N were fixed for alleles from group A. These northern accessions also displayed greatly reduced *SGR* sequence diversity compared to group A accessions collected from other regions, suggesting that the northern populations may represent recent extensions of the range of the species. Group B accessions were distributed from Lake Tiberias south and were generally sympatric with the southern group A accessions. Although group B accessions occupied a smaller area than group A, the SGR sequence diversity in this group (28 alleles in 33 accessions) exceeded that for group A.

## Introduction

*Pisum fulvum* Sibth. & Smith is a wild species that is easily distinguished from other *Pisum* taxa by its pale yellow to orange yellow flowers (Ben-Ze’ev and Zohary, 1973) and short pods that often exhibit a tendency towards amphicarphy (Hellwig et al., 2020a). Presently, it is widely accepted as one of two species in the genus, the other being the wider ranging and more genetically diverse *Pisum sativum* L. (Maxted and Ambrose, 2001; Hellwig et al., 2021a). The status of *P. fulvum* as a distinct species has been rarely questioned because in addition to the marked morphological differences between it and *P. sativum*, there exists a strong fertility barrier, with hybrid seed being difficult to generate and usually producing F_1_ plants with greatly reduced fertility (Kosterin et al., 2019).

Recent studies involving an extensive collection of *P. fulvum* from Israel (Naim-Feil et al., 2017, Hellwig et al. (2020b; 2021a; 2021b) revealed that this species is genetically less diverse than *P. sativum*, and appears to have gone through a more severe reduction in its former diversity. Interestingly, these authors found only a weak correlation between ecological factors and genotype, implying that isolation by environmental differences was not a primary factor in the partitioning of genetic diversity. Hellwig et al. (2021a) provided evidence that considerable outcrossing occurred in *P. fulvum*, and suggested that cross pollination may be partly responsible for the lack of correlation between genetic partitioning and environmental variation.

In a previous study involving a sample of 137 *Pisum* accessions focused primarily on the wild *Pisum sativum* subsp. *elatius* (M. Bieb) Asch. & Graebn. germplasm, we described a hypervariable region of the *SGR* (*STAYGREEN*) locus (Mendel’s cotyledon color gene) and used this DNA fragment to distinguish accessions of the subspecies and reveal phylogenetic relationships among the accessions (Weeden et al., 2021). In that previous study we also identified seven *SGR* alleles in ten accessions of *P. fulvum*, suggesting that the DNA fragment may also be hypervariable in this latter species. In addition, the seven *P. fulvum* alleles appeared to split into two primary groups, one diverging at the base of a Bayesian phylogeny of the genus, and one appearing to be derived within *P. sativum* sequences. As other phylogenetic analyses using DNA sequences have generally found that *P. fulvum* germplasm sorts out as a monophyletic assemblage (Jing et al., 2007; Smýkal et al., 2017; Liu et al., 2022), a much more extensive analysis of SGR sequence diversity in *P. fulvum* seemed appropriate.

The set of *P. fulvum* accessions examined by Naim-Feil et al. (2017) provided excellent material to expand our analysis. Specifically, we intended to address four questions: (1) does *P. fulvum* have significantly less diversity at this DNA segment than *P. sativum*, (2) does SGR variability continue to separate *P*. *fulvum* into two clades, (3) does the SGR variability provide evidence of hybridization within the genus, and (4) do SGR subclades of *P. fulvum* accessions correlate with ecological, geographical, or phenotypic parameters?

## Materials and methods

### Plant material

The 90 accessions of *Pisum fulvum* examined and, when known, their respective collection sites are listed in **Supplemental Table S1**. Accessions with an "L" prefix were obtained from Dr. Giles Waines, U.C. Riverside, those with "Pf" designation were obtained through the USDA from material collected by S.A. When available, preliminary USDA accession designations (W6 numbers) are given for these accessions in **Supplemental Table S1**. Additional accessions of *P. fulvum* were obtained from the USDA collection under their PI number. Two samples of *P. fulvum* from Israel (VIR 6070 and VIR 6071) were kindly supplied by Dr. F. Gorel, then at the Institute of Cytology & Genetics, Novosibirsk, Russia. The two final *P. fulvum* accessions (JI 2204 and JI 2205) were obtained from the John Innes Centre, Norwich, UK. Passport data for these can be obtained in Naim-Feil et al. (2017) or on the GRIN website (npgsweb.ars-grin.gov/gringlobal/search). The 90 accessions included eight pairs (PI 595947/VIR2523, PI595948/VIR3397, JI2205/VIR6070, PI560064/L95, PI 433560/W6 15045, PI560066/L97, PI 560063/L94, and PI 595953/VIR6071) that, based on passport data, were the same accessions but had been assigned different designations at different institutions. These pairs were analyzed independently as internal checks of our methods.

Only 55 of the accessions examined by Naim-Feil et al. (2017) were analyzed in the current study. The accessions were selected to represent the complete geographical range of the collection with at least one sample taken from every genetic cluster identified in Hellwig et al. (2020b).

The 63 P*. sativum* SGR sequences included in the analysis were selected to include each of the alleles reported in Weeden et al. (2021). ‘Delta,’ ‘Dakota,’ and ‘Melrose’ are commercial varieties (*P. sativum* subsp. *sativum* L.) common in the United States, the first two typically being grown as dry peas and the last categorized as an Austrian winter pea (Auld et al., 1978). WL808 is a widely studied representative of *P. sativum* subsp. *abyssinicum* (A. Br.) Govorov. The remaining represent either landraces of *P. sativum* subsp. *sativum* or accessions of *P. sativum* subsp. *elatius* (M. Bieb.) Asch. & Graebn.

Accessions were grown in the greenhouses at Montana State University during fall (September through December and spring (February through June) seasons. Supplemental lighting with high pressure sodium lights (SON AGRO 430 watt, Philips Lighting Co., Somerset, NJ) was used to extend the daylength to 16 hr. Temperature was maintained between 18 and 25° C. Plants were grown in individual 12 cm pots containing a 50% peat, 25% sand, 25% local soil mix.

### Morphological characterization

All *P. fulvum* lines were characterized for six morphological characters (**Supplemental Table S1**). Each accession was grown at least twice, once in the spring and once in the fall. Several of these traits (testa color, testa markings, axil pigmentation, and leaflet serration) show similar variation in *P. sativum* and in the latter species exhibit a simple genetic basis (Blixt, 1972). The morphological traits in *P. sativum* (and controlling genes) were brown mottle on seed coat (*M*), violet markings on seed coat (*Fs,U* and *U^st^*), axil pigmentation (*D*), and leaflet serration (*Ser*).

### Sequence targeted

The amplicon analyzed in this study was intron 3 of the gene responsible for the green/yellow cotyledon color polymorphism described by Mendel (1866), designated *I* by White (1917) and renamed *STAYGREEN* (*SGR*) by Armstead et al. (2007). The gene resides on chromosome 2 of the pea reference genome (https://www.ncbi.nlm.nih.gov/datasets/genome/GCF_024323335.1/) position 482796788 to 482788825. The DNA sequence compared in the present study was that between 482796060 and 482789149. The forward primer used to amplify this sequence was the same as in Weeden et al., 2021, whereas the reverse primer was changed to 5’-ATTGCCGTCACCGTGAAC in order to slightly shorten the fragment amplified, excluding about 100 bases of flanking exon sequence that exhibited low variability.

### PCR conditions

All PCR reactions (20 ul) contained the following: 4 ul Promega 5X PCR buffer, 2.5 mM MgCl_2_, 0.3 uM of each dNTP, 0.6 units Promega *Taq* polymerase, and usually 10 to 30 ng pea genomic DNA. Forward and reverse primers were added at a concentration of 0.6 uM. For most PCR amplifications a touchdown procedure was employed, with annealing temperature starting at 63°C and dropping in 1° steps to the final annealing temperature of 57°C. After the touchdown portion the PCR was continued for 30 cycles. Initial denaturation at 92°C was 2 min. Successive denaturing steps were for 30 sec. A 45 sec annealing time and 90 sec extension time was used. In a very few instances, duplicate samples were run using a higher template DNA concentration (approximately 100 ng) and amplification was performed without the touchdown portion of the procedure, instead going to a 57°C annealing step immediately after the initial denaturation. In addition, only 26 cycles were completed during these amplifications.

### DNA sequencing

Forward and reverse sequences were obtained through Sequetech (Mountain View, CA USA) as described in Weeden et al. (2021). All sequences mentioned in the current study can be found in FASTA format at the PulseDB website (https://www.pulsedb.org/PubDatasets) and in an aligned format in **Supplemental Table S2**.

### Alignment of sequences

The raw SGR sequences were trimmed slightly, confirmed by visual inspection of the trace supplied by Sequetech, and then aligned by three different methods. Initial alignment was performed using MAFFT (MAFFT alignment and NJ/UPGMA phylogeny (cbrc.jp). A second alignment was generated using multiple refinement iterations of MUSCLE (Edgar, 2004a; Edgar, 2004b). This process included MUSCLE refinement of MAFFT aligned sequences, in addition to an initial MUSCLE alignment followed by multiple refinements until alignments stabilized. Because of the considerable number of repeats in the sequences, a third alignment was done manually. It is well known that short simple sequence repeats (SSRs) occur frequently in eukaryotic genomes as a result of a strand slippage mechanism (Eckert and Hile, 2009), and are often present in a head-to-tail arrangement. As other head-to-tail repeats can arise by a similar unequal crossover mechanism, for the manual alignment we placed a priority on keeping the highly homologous repeats in a head-to-tail arrangement, assuming they arose recently. However, sets of repeats that exhibited significantly lower homology between sets than within were treated as having arisen on different occasions and were not maintained in tandem if alternative alignments gave higher similarities among sequences. The manual alignment also prioritized sequence homology over alignment length. That is, if one alignment gave a higher base match percentage and another produced a slightly shorter overall alignment, the former alternative was usually selected.

### Phylogenetic analysis of DNA sequences

We analyzed alternative DNA sequence alignments using MrBayes 3.2.7a (Huelsenbeck and Ronquist, 2001; Huelsenbeck et al., 2001). Nucleotide sequence data were analyzed during Bayesian runs by estimating a gamma distribution, proportion of invariant sites, and a nucleotide substitution model using the “mixed” option. Each run included four MCMC chains each with 4 M generations and sampling every 4 K generations. We used the default 25% burnin for generating a Bayesian consensus tree and the “sump” option to ensure sampling was performed at likelihood stationarity.

To further investigate nucleotide substitution rate and evidence for homoplasy we analyzed sequence alignment alternatives using BEAST v.2.6.7 (Bouckaert et al., 2014, http://www.beast2.org/), which integrated the choice of nucleotide substitution models, molecular clock models, and tree (speciation) models. Nexus files of the alternatively aligned *SGR* sequences were imported into BEAUti v.2.6.7. The site model involved model jumping using the Beast Model Test. A relaxed log normal molecular clock (Drummond et al., 2006) was selected. For the tree model, we used the Yule model because it estimated faster substitution rates than the birth-death and other models and we want to bias for fast substitution rates. We implemented tree priors so as to root trees using the *Pisum sativum* accession W6-26109 as the outgroup. Using estimates from Lavin et al. (2005) and Weeden et al. (2021), we set the mean age for the root of the tree as a young age at 5 Ma in order to bias for faster substitution rates. We ran a Monte Carlo Markov Chain (MCMC) for 50 million generations and sampled parameters every 50,000 generations. Using parameter and tree samples derived from each alternative DNA sequence alignment, Tracer v.1.6 (Rambaut and Drummond, 2013) identified the burn-in and summarized parameter estimates at likelihood stationarity. TreeAnnotator v.2.6.7 output the Bayesian maximum clade credibility (MCC) tree with median ages and 95% highest posterior density (HPD) intervals of node ages from each BEAST2 analysis using a 10% burn-in.

For the parsimony analysis using PAUP (Swofford, 2003), we conducted a heuristic search, which included 100 random addition replicates, treebisection-reconnection, and retention of multiple parsimonious trees. A maximum of 10,000 trees was allowed to accumulate. Bootstrap analysis and partition homogeneity tests involved re-sampling with replacement (Felsenstein, 1985; Sanderson, 1995), where 10,000 replicates were each subjected to random addition of taxa, tree-bisection-reconnection, and invoking neither steepest descent nor retention of multiple parsimonious trees. Although we analyzed multiple alternatively aligned SGR sequence data sets, we report only the results that were consistently resolved across all sequence alignments and that were relevant to the questions addressed in this study.

## Results

SGR sequences were obtained for 90 P*. fulvum* accessions, of which 82 were believed to be non-synonymous. Among these, 57 distinct alleles were identified (**Supplemental Table S1**). The percentage of unique alleles in *P. fulvum* (57/82 or 69.5%) was slightly less than what we had found previously for wild *P. sativum* (44/51 or 86%); however, this percentage still represented an extraordinarily high amount of sequence polymorphism in a short fragment of DNA. In general, the sequences from the *P. fulvum* accessions were shorter (529 to ~1100 nt) than those previously found for wild *P. sativum* accessions (904 to 1306 nt). The several duplicate samples that were amplified using greater concentrations of template DNA and a cycle sequence that omitted the touchdown portion produced amplified fragments of the same size and sequence of those fragments produced by the standard cycle. This consistency between duplicates indicated that at least most of the variation was not caused by PCR artifacts generated by the repeats present in the amplified fragment.

**Figure 1:**
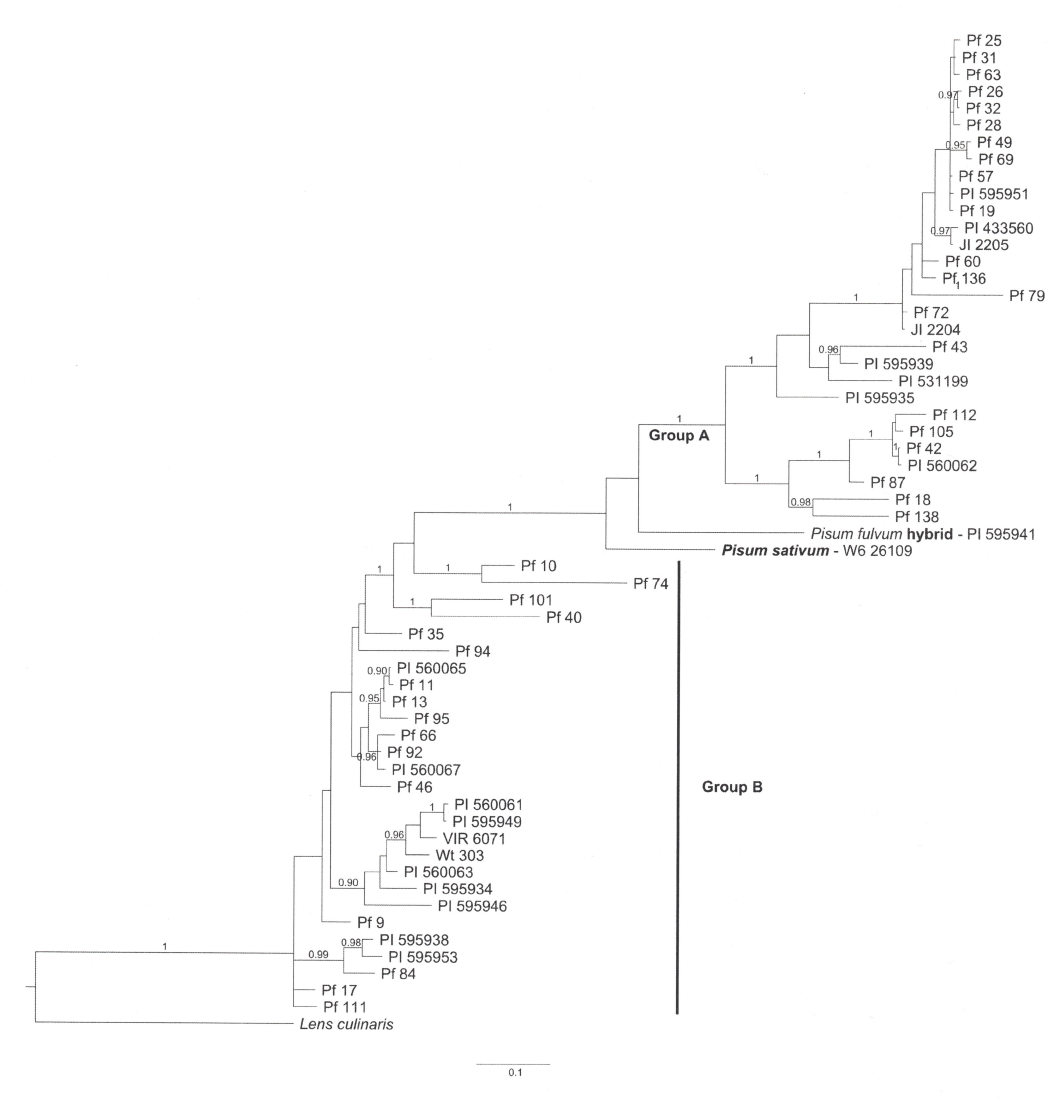
Bayesian tree for 57 intron 3 sequences of the *STAYGREEN* gene in *Pisum fulvum*. The homologous sequence in *Lens culinaris* was used to root the tree, and the *Pisum sativum* sequence for W6 26109 (in bold) indicates where most sequences from that species would be placed if included. The Group A label identifies the clade containing all *P. fulvum* group A sequences. The Group B label delineates the polyphyletic (in this analysis) assemblage of group B sequences. PI 595941, the *P. fulvum* accession suspected of being relatively recently derived from a *P. fulvum* x *P. sativum* hybridization can be observed adjacent to W6 26109.

The large number of indels, particularly tandem repeats (perfect and imperfect), complicated the alignment process. The three alignments used in this study (those generated by MAFFT, MUSCLE, and manually) all differed in numerous sections. For the manual alignment the priority for maintaining highly homologous repeats as tandem blocks created relatively large gaps when aligning with sequences from other accessions. For this reason, the manual alignment was longer (nearly 6000 positions, including gaps) than those of the other two alignments (1958 nt for MAFFT and 3437 for MUSCLE refined MAFFT). However, each of the alignments generated a similar Bayesian tree. The Bayesian tree generated from the MUSCLE alignment is shown in **Figure 1**.

For reference, the manual alignment including all *P. fulvum* and *P. sativum* alleles is included as **Supplemental Table S2**. Because this alignment includes all the *P. sativum* alleles rather than just the two outgroups (*Lens culinaris* and *P. sativum* W6-26109) used in the Bayesian analysis of *P. fulvum* sequences, it was longer (nearly 8000 nt) than the manual alignment generated for just the *P. fulvum* analysis. However, except for the additional gaps, both alignments are similar, and **Supplemental Table S2** can be used as a reference for both the *P. fulvum* analysis and the general *Pisum* analysis described below.

In each alignment, the first approximately 80 bases of sequence at the 5’ end showed high conservation, with only the number of T’s around position 50 and indels starting at position 70 producing significant variability (**Supplemental Table S2**). Similarly, the sequence of approximately 130 bases at the 3’ end of the sequence was highly conserved in *P. fulvum* accessions (**Supplemental Table S2**), with only Pf 84 displaying an apparent deletion extending into this region. Between these two end regions numerous indels and various repeats were present. There were many cases of a short (3 to 15 nt) sequence being repeated once in a head-to-tail arrangement. Less common were tandem repeats, either perfect or slightly modified, occurring more than twice. These tended to be longer (20 to 60 nt) in the size of the repeat unit and were often limited to a subset of accessions. There was also a major repeat structure to the section of the intron between the 5’ conserved region and the last 50 nt of the 3’ conserved region. In **Supplemental Figures S1, S2** the indel sequences for a selected set of *P. fulvum* accessions are formatted in a way to emphasize this major repeat structure. In the figure the sequence for each accession is broken so that nearly every line, except the first, contains a full or partial repeat unit. Near the start of most lines can be found a CTCTTCTAAT or GTAAGTCTAAT motif. Near the center of each line can be found a GA-rich segment to be described below. Near the end of many of the lines is a sequence similar to CTTGTTTGTGTCTGGT. This general arrangement, with many deviations, is repeated within the indel 5 to 20 times.

### Morphological observations

The morphological traits examined proved not to be as consistent as their counterparts in *P. sativum*. The testa colored varied from tan to solid dark violet (appeared black). Seed produced from the same plant would occasionally vary in testa color, although the lighter seeds often were smaller and probably matured later. The dark, solid testa color present in many accessions prevented assessment for the presence of spots or marbling, and in such cases the latter traits were scored as ‘unknown’ (**Supplemental Table S1**). Even a brown testa often obscured the brown marbling trait, as did the intense violet dotting phenotype characteristic in a few of the accessions. Thus, the brown marbling trait was of little value as a diagnostic character.

The red pigmentation in the leaf axil also displayed inconsistent expression. We have observed *P. fulvum* lines with a very intense pigmentation extending from the stem/stipule contact outward 1-2 mm (unpublished observations). However, in the greenhouse nearly all the plants we observed either lacked axil pigmentation (or with a barely detectable pigment line right at the stem/stipule contact) or possessed a ring of dots on the stipule 1-2 mm from its base (the dots sometimes becoming intense enough to fuse together to form an incomplete ring of color). The variation in axil coloration was best observed on stipules on nodes 4-6. Occasionally, the base of the leaflets or the base of the sinuses between deep serrations would be pigmented. These phenotypes were nearly always associated with the presence of red pigmentation on the stipule.

The violet spotting or streaking on the pods also displayed considerable variation in intensity which we concluded was due to incomplete expression or varying environmental factors because under certain conditions some clear pods would be produced on the same plant producing pods with the flecked or hazy purple phenotypes (unpublished observations). Plants from accessions identified as having the ‘strongly flecked’ phenotype in **Supplemental Table S1** generally produced a majority of pods with strong flecking. In addition, there were some accessions that never produced spots on the pods. However, we could not establish reliable categories within the faintly spotted (sometimes not spotted) to clearly spotted continuum.

The most reliable of the morphological characters we assessed was serration of the leaflets. This trait varied from leaflets being essentially entire to nearly pinnately 6- to 8-lobed. Repeated examination of *P*. *fulvum* in the greenhouse over several years indicated that accessions can be separated into those with entire or weakly serrate leaflets, those with clear leaflet serration (‘dentate’ in **Supplemental Table S1**), and those with strongly serrate to lobed leaflets. In **Supplemental Table S1**, four phenotypes are listed for leaflet morphology (entire, weak, dentate, and strong), but distinguishing between the entire and weak categories was unreliable.

### Characterization and comparison of SGR sequences in *P. fulvum*

The variation observed in the SGR fragment in *P. fulvum* was easier to characterize than that in *P. sativum* reported previously (Weeden et al., 2021) for not only was the amplified fragment generally shorter but also the repeat structure was more conserved. Excluding the sequence for PI 595941, *P. fulvum* sequences partitioned into two groups, designated A and B (**Supplemental Table S1** and **Figure 1**). All 90 P*. fulvum* accessions are presented in **Supplemental Table S1**, whereas only representatives of the 57 different alleles are included in **Figure 1**. The affinities of the PI 595941 allele will become clear when *P. sativum* sequences are included in the analysis (see below), and for the present we will ignore this sequence. Each of the groups could be further divided into subgroups based on sequence similarities, particularly presence or absence of indels (**Supplemental Table S1**).

A few short sequence motifs were commonly found in these sequences and proved helpful in understanding their structure. Most of the repeats contained a GA-rich region with a sequence approximating 5’-AGAAGAGGAGGAGAGA. Occasionally this sequence was extended by a 10-15 base addition similar to 5’-CAAGAGGATAAA. These motifs will be referred to as ‘GA-rich’ and ‘GA-rich extension,’ respectively. Repeat units containing such sequences will be referred to as ‘GA-rich repeats.’ A second useful feature was the appearance of a 5’-GTAAGTCTAAT sequence or slight variants thereof. In this paper it will be referred to as the ‘GTAAGT motif.’ Finally, two closely spaced poly-T sequences (positions 50 to 61 and 71 to 76 in **Figure S1**) were helpful for distinguishing some of the subgroups. The first consisted of 7 to 11 T’s and will be referred to as the ‘poly-T sequence.’ The second was preceded by a C and usually consisted of 6 T’s. It is referred to as the ‘CTTTT sequence.’

Group A included the shortest sequences and exhibited the simplest repeat structure. Based on indel structure and, to a minor extent, plant phenotype, this group was divided into six subgroups, designated A-1-a, A-1-b, A-2, A-3, A-4-a and A-4-b (**Supplemental Tables S1**, **S2**). Within group A-1-a accessions, four SGR alleles were present. Three of these alleles possessed identical lengths (536 nt) and differed only by 1 or 2 single nucleotide polymorphisms (SNPs) (**Supplemental Tables S1, S2**). The fourth (Pf 136) differed from the others by a small deletion and two insertions, all of which were unique to this accession (**Supplemental Table S2**). The overall structure of the A-1-a sequence consisted of relatively conserved ends, an interior region with four imperfect repeats, each containing a GA-rich segment with that of the fourth possessing the GA-rich extension. The first of the repeats had a 31-base duplication at its 3’ end. Most of a fifth imperfect repeat, as well as a partial sixth were included in the more conserved 3’ end of the fragment (**Supplemental Figure S1**). All members of this group shared a 6-base deletion in the CTTTT sequence (in the conserved 5’ portion) with sequences from A-1-b, A-2 and A-3 (**Supplemental Figure S1** and **Supplemental Table S2**).

The set of accessions with the 536a allele fell into two morphological groups. The first four non-synonymous accessions in **Supplemental Table S1** possessed olive testa, displayed dentate or lobed leaflets and produced a purple hue on most of their pods. In contrast, the remaining four non-synonymous accessions with this allele had a darker testa, more or less entire leaflets, and for the two that produced pods, lightly spotted pods. The uniformity of the first four accessions was remarkable, particularly because the purple haze phenotype was relatively rare among the accessions we investigated. It is possible that they could all be synonymous or collected from the same population. Most of the A-1-a accessions were collected south of Haifa (at least for those which such passport data was available). Only Pf 24 and Pf 29 were collected near the Lebanese border.

Subgroup A-1-b sequences were very similar in structure to the A-1-a sequences except that the first two GA-rich repeats are duplicated, producing a total of eight GA-rich repeats in the fragment (**Supplemental Figure S1**). As was the case for the A-1-a sequences, most of the sequence variation in A-1-b consisted of SNPs. The Pf 49 sequence differed by only a single base deletion in the 3’ conserved region and the 697 alleles are generated by a GAG insertion in a simple sequence repeat (SSR) region. Even the much larger fragment amplified for Pf 79 was very similar to the standard A-1-b structure except that it contains a novel 176 base GA-rich sequence that was repeated once in a head-to-tail arrangement (**Supplemental Table S2**)

In contrast to most of subgroup A-1-a, most of the A-1-b accessions were collected from the extreme north of Israel along the Lebanese border (**Supplemental Table S1**). Their morphological phenotypes resemble those of the second set of A-1-a accessions (dark testa, entire leaflets, spotted or flecked pods). Leaf axil pigmentation covered a range of phenotypes, with two (Pf 63 and Pf 79) having red pigment at the base of the leaflets as well at a ring of dots in the axils). Exceptions occurred for this general morphological characterization, but particularly remarkable exceptions were the strongly serrate leaflets of Pf 19 and Pf 28 (**Supplemental Table S1**). The four accessions in this category collected farther south (Pf 49, Pf 79, Pf 116, and Pf 120) were not particularly distinctive either individually or as a group.

Subgroup A-2 sequences were intermediate in length between those from groups A-1-a and A-1-b (**Supplemental Table S1**). The common two alleles (length 607 nt) have one additional GA-rich repeat (without extension) in the interior portion of the amplicon, as well as a duplication of an AGTCT segment within the fourth GA-rich repeat (**Supplemental Figure S1**). Except for W6-15045 and Pf 60, A-2 accessions had entire leaflets and the pods usually had violet dots on their surface. The testa was tan or dark, often exhibiting a brown mottle when the testa was tan. Although collection site data were limited for several of the accessions in this group, most of the accessions for which data were available were collected just west of Jerusalem.

The outlier, Pf 60, displayed a somewhat disrupted repeat pattern, with several apparent deletions as well as showing certain affinities to sequences in subgroup A-3. The strong leaflet serration observed in this accession might indicate an association with Pf 19 (allele 694a) or the set of accessions with allele 536a and strongly serrate leaflet. However, the phenotype for Pf 60 is a poor match for the other traits of Pf 19 or the 536a set, and it was assigned to A-2 based on its possession of one GA-rich extension and lack of a GTAAGT motif. It was collected west of Jerusalem.

Two pairs of lines, supposedly representing duplicate accessions (JI2205/VIR6070 and PI 433560/W6-15045) displayed variation in testa phenotype. JI2205 and VIR6070 both showed within line variation during multiple plantings. Similarly, PI 433560 and W6-15045 (the former an updated designation for the latter) displayed this same variation in testa color, although in this case PI 433560 had variation within the accession, whereas W6-15045 was uniformly tan. Of even greater concern was the observation of entire leaflets on PI 433560 plants but strongly serrate leaflets characterizing W6-15045 plants. Such a major difference would indicate mislabeling of one or the other accession. Strongly serrate leaflets were not otherwise present in this group.

The sequences in subgroups A-3 and A-4 were more diverse than in the previous subgroups, and some short sequences not found in other *P. fulvum* accessions occurred in some members of both A-3 and A-4. Thus, the splitting of the two subgroups was somewhat arbitrary. However, except for the A-4-b accessions, both subgroups possessed a single GTAAGT motif (**Supplemental Figure S1**), being the only group A subgroups containing this sequence. Note that all A-3 and A-4a and b sequences had slight variants of the GTAAGT motif (highlighted in gray in **Supplemental Figure S1**). Similar or further modified motifs are present in A-1 and A-2 sequences. In the current study, subgroup A-3 sequences were defined by a lack of the CTTTT sequence, the presence of one GTAAGT motif, and the lack of a TATYCG combination (position 119-124 in **Supplemental Table S2**) found in all other group A sequences. In addition, all subgroup A-3 members displayed a 24 base indel (position 7605 to 7631) unique to group A sequences and all except PI 595935 had a unique 47-base sequence repeated three times (position 5043 to 5192 in **Supplemental Table S2**). Subgroup A-4 sequences had both the CTTTT and TATYCG sequences, and instead of the 47-base repeat found in group A-3, possessed a unique 65-base (subgroup A-4-a) or a 39-base (subgroup A-4-b) insert (position 912 to 977 in **Supplemental Table S2**).

In the greenhouse, plants from both subgroups typically had entire to slightly serrate leaflets (PI 595935 and Pf 105 were exceptions) and faint dots of red pigment in the leaf axils (again PI 595935 was an exception). Indeed, PI 595935 could be considered an outlier to both A-3 and A-4 subgroups and was the only accession examined identified as being collected from Syria. It also was the only accession in the group with strongly serrate (almost lobed) leaflets, and red pigmentation was apparent not only in the leaf axils but also in the leaflet axils and occasionally on the surface of the leaflets.

We examined the A-3 and A-4 accessions for correlations between any allele(s) and other characteristics of these accessions, but very few correlations were discerned. Two (PI 595939 and PI 595940) of the three accessions possessing the 918 nt allele were very similar in phenotype and could possibly be synonymous or closely related. The third accession with that allele, Pf 80, differed considerably in morphology as well as collection site. The other A-3 accessions gave no clear grouping with respect to morphology or collection location (**Supplemental Table S1**). Similarly, in subgroup A-4 only two correlations of interest were found. The two A-4-b accessions (Pf 18 and Pf 138), which formed a terminal branch in our Bayesian analysis, displayed a very similar morphology, including the relatively rare intense violet spotting of the testa. The two accessions were collected very near each other and were placed very near each other in the tree developed by Hellwig et al. (2020b). Similarly, Pf 105 and Pf 112 formed a terminal branch both in our analysis and in that of Hellwig et al. (2020b), despite displaying different morphologies and collection sites. However, in general the relationship between the postulated phylogeny of the *SGR* alleles and similarity of phenotype was very obscure.

The repeat structure of group B sequences differed markedly from those of group A. Our manner of formatting sequences based on GA-rich sequences did not expose the repeat structure as successfully in group B sequences because group B repeats occasionally displayed two GA-rich regions within the major repeat (**Supplemental Figure S2**). For instance, in subgroup B-5 the combined fifth and sixth lines constitute a fragment that is imperfectly repeated (a third GA-rich repeat is present as line 8) in lines seven through nine (**Supplemental Figure S2**). As is evident in **Supplemental Figures S1, S2**, the number of GA-rich repeats in group B sequences (except for group B-5) is greater (13-18) than that in group A (6-13). In part, the greater number of GA-rich repeats in group B was responsible for the longer average length of the fragment amplified (817 nt in group B, 745 nt in group A), but there were additional qualitative differences that separate the sequences of these groups. These differences include 17 significant indels summarized in **Supplemental Table S3**, and indicated a much more fundamental divergence between the two groups than just number of GA-rich repeats.

The most striking of these differences was the prevalence of the GTAAGT motif in group B sequences. In group A, this motif was found only in sequences from the A-3 and A-4 subgroups, and only once per sequence, although slight modifications of the motif were present as well. In group B the motif occurred multiple times in every sequence. Allowing for minor variants in the motif (highlighted in gray in **Supplemental Figures S1, S2**), there appeared to be one GTAAGT motif for nearly every major repeat unit, and the presence of the sequence was helpful in determining if the repeat had one or two GA-rich sequences. A second difference in structure was the absence of any GA-rich extensions in group B sequences, whereas every group A sequence possessed such a region. Despite the significant differences between group A and group B sequences, there were no extensive sequences distinguishing the two groups when the sequences were aligned using MUSCLE or MAFFT. Only in the manual alignment did significant differences become apparent between the two groups (**Supplemental Table S2**). Group A clearly lacked a short fragment (position 247-270) present in not only group B sequences but also most *P. sativum* sequences (**Supplemental Table S2**). This fragment was part of a complex repeat with units distributed from position 110 to 510.

The next major difference (from position 1183 to 1324), present in group A but not group B, is a tandem duplication (more or less) of the sequence preceding it that is present in both groups. Interestingly, much of the duplicated segment is also present in most *P. sativum* sequences (**Supplemental Table S2**). The sequence fragment between positions 1645 and 1680 is merely an additional duplication of the previous segment that appears to be unique to group A. The fragment between position 1838 and 1920 that is lacking in group A is the first appearance of the GTAAGT motif. This fragment is also present in *P. sativum* in the alignment shown in **Supplemental Table S2**. The second GTAAGT motif appears in many group B sequences at position 3918 (**Supplemental Table S2**). A fragment of subgroup A-1-b sequences partially overlaps the GTAAGT motif, but the motif is degraded and split in the latter subgroup, or perhaps the sequences are misaligned at this point. This GTAAGT motif also appears to have an identical homolog in several *P*. *sativum* sequences (**Supplemental Table S2**). The two segments (position 4950 to 4984 and 5659 to 5742) present in group A sequences but lacking in group B and *P. sativum*, represent additional copies of sequences at least partially present in all *SGR* sequences. It was difficult to determine just where these sequences best fit into the alignment in **Supplemental Table S2**, and thus their precise positions should not be given much significance. Finally, the last three relatively short fragments found in group B (**Supplemental Table S2**) contain repeat sequences present in other regions of most *SGR* amplicons. The one somewhat unique and useful aspect of the sequence between positions 5827 and 5851 is that it contains a *Dra*I restriction site (starting at position 5885) not present in group A or *P. sativum* sequences. This restriction site could be used as an identifier for most group B sequences. Another restriction enzyme useful for distinguishing the two groups was *Hin*dIII. All subgroup B-6, B-7, and B-8 (except the B-8 ‘outsider’ Pf 101) sequences had a single *Hin*dIII restriction site 95 nt from the 5’ end of the tailored sequence, while none of the Group A sequences were cut by the enzyme.

**Figure 2:**
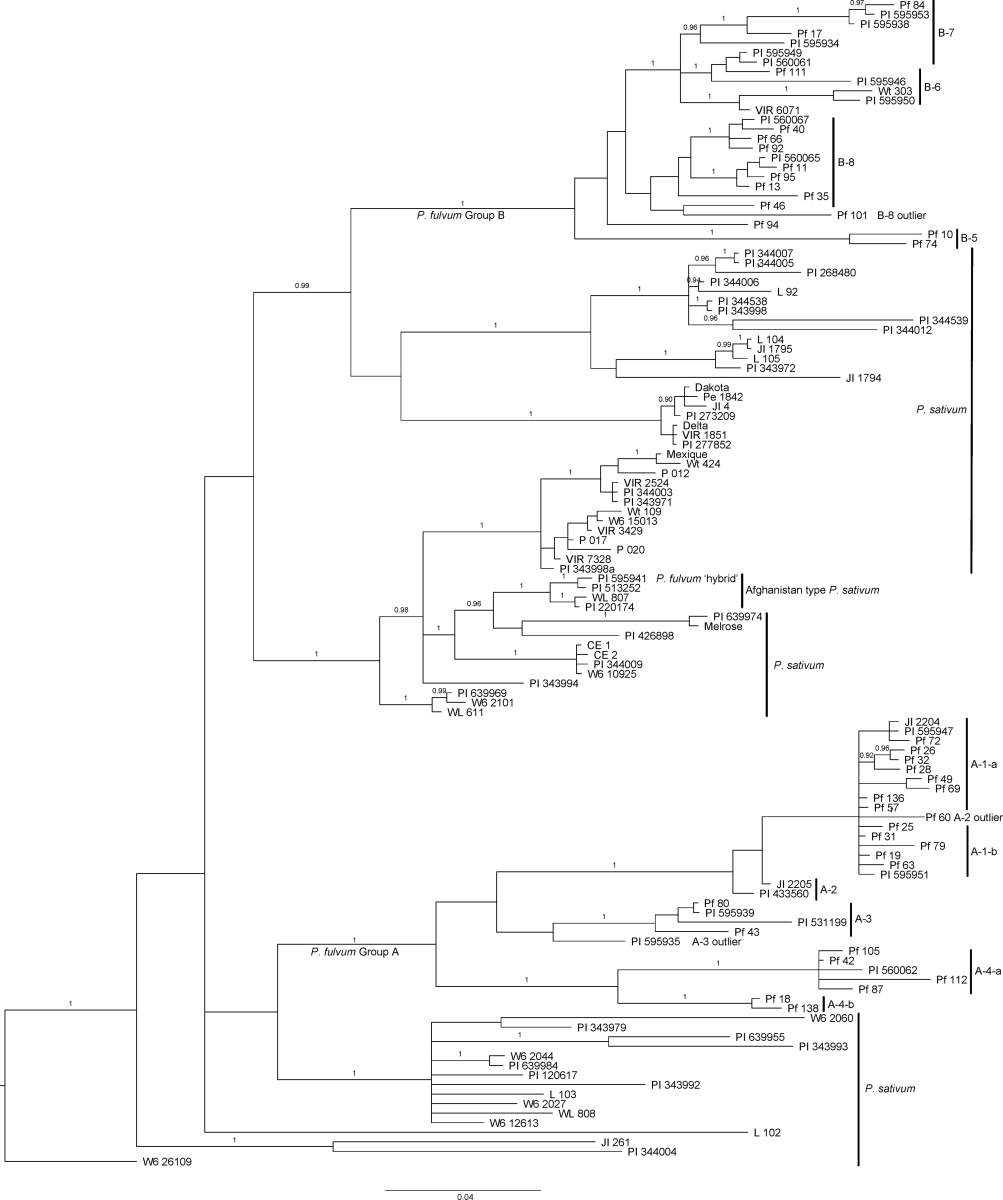
Bayesian tree for intron 3 sequences of the STAYGREEN gene in *Pisum* depicting associations among all alleles identified in the genus. The tree was rooted with the sequence from the *P. sativum* accession W6 26109. Subgroups of *P. fulvum* accessions are labeled as in **Supplemental Table S1**, with all group B sequences forming a monophyletic clade at the top of the figure and group A sequences forming a monophyletic clade branching in the lower half of the figure. The sequence from PI 595941, the *P. fulvum* accession suspected of being relatively recently derived from a *P. fulvum* x *P. sativum* hybridization, clusters with those of three *P. sativum* accessions labeled as Afghanistan type.

The group B sequences could be separated into at least four subgroups (B-5, B-6, B-7, and B-8 in **Supplemental Tables S1**, **S2**). As was the case for the A-3 and A-4 grouping, there was considerable sequence variation within each of the B subgroups, making our divisions somewhat arbitrary. However, in general, the subgroups correspond to major branches in the Bayesian phylogenetic analysis (**Figure 2**) and were helpful for comparing our results with previous results on the same accessions.

For each of the three trees generated from the three different alignments, subgroup B-5 was the basal branch of the group B clade (**Figures 1**, **2**). However, its two members, Pf 10 and Pf 74, had relatively divergent sequences, suggesting they were not closely related. What appeared to hold the sequences together was that they both possessed a poly-T region longer than 10 bases and a unique deletion between positions 264 and 530 (**Supplemental Table S2**). In contrast, subgroup B6 sequences were relatively uniform, and shared a unique multiplication of a 43 nt fragment (position 401 to 510 in **Supplemental Table S2**), with VIR 6071 and PI 595943 containing three and a half copies of this sequence, whereas Wt303 and PI 595950 only displaying two and a half copies. All four also had a unique 6-base tandem duplication starting at position 1047 and a unique 8-base duplication starting at position 1889. The indel arrangement for PI 595950 was identical to that of Wt303, suggesting that the two could represent synonymous accessions. Passport data for these two accessions was incomplete, and both sequences had ambiguous base calls in specific regions. However, two of the SNPs appeared real and the accessions differed in their axil color phenotype, so they have been treated here as non-synonymous. The other pair of accessions in this subgroup are identified by passport data as synonymous and display identical SGR alleles and similar phenotypes (**Supplemental Table S1**).

Subgroup B-7 sequences all shared a 29-base deletion starting at position 71 that included all T’s in the CTTTT sequence. The remaining portions of these sequences were relatively similar, although a variety of indels produce eight fragment lengths among the 13 non-synonymous accessions. We split the subgroup into two further subdivisions because three of the sequences (Pf 84, PI 595938, and PI 595953) appeared to be very closely related, and two of these were the only accessions in B-7 with strongly serrate leaflets (**Supplemental Table S1**). Axil pigmentation in the subgroup was mostly the red dot phenotype (Pf 5 showed an absence of axil pigmentation and PI 595934 showed and intense ring of pigment). However, testa color and pattern differed widely, as did collection site, indicating little correlation between morphology or geography and similarity of the SGR alleles.

In two cases within subgroup B-7 non-synonymous accessions shared the same sequence (PI 595933/PI 595934 and Pf 9/Pf 73). Phenotypes of the paired accessions were relatively similar and distinctive. The PI 595933/PI 595934 pair had light testa with many violet spots, as well as spotting on the pod. This combination of traits was uncommon, suggestive of a close relationship between the two accessions. Similarly, Pf 9 and Pf 73 possessed similar phenotypes except for testa color. However, the collection sites for this latter pair differed considerably (west of Jerusalem versus just northwest of Lake Tiberias).

Subgroup 8, including the outliers Pf 94 and Pf 101, contained 13 accessions. All had relatively standard B sequences that differed for many indels, producing 13 different alleles. Four accessions (Pf 40, Pf 66, Pf 92 and PI 560067) all shared a 9 base imperfect tandem repeat (position 543) not present in any other Group B accession. However, these four differed for other indels (Pf 66 had a very large deletion starting at position 1900, and Pf 40 had three additional imperfect copies of a tandem repeat starting at position 4250), as well as for morphological traits and geographical origin. There were no indels shared by all members of this subgroup and absent in other subgroups. Thus, subgroup 8 was basically a collection of accessions that did not fit any of the previously defined subgroups.

When group A and group B sequences were compared for relative amounts of diversity within each group, group B appeared to possess the greater variation. Although there were more alleles identified among the group A accessions examined (29 alleles in the 48 accessions believed to be non-synonymous compared to 28 alleles in the 33 non-synonymous group B accessions), many of the group A alleles differed by only one or a few SNPs, whereas most of the group B sequences differed by one or more indels (**Supplemental Table S1**). If alleles that differed only by SNPs are combined, group A would have only 19 alleles, whereas group B would contain 26. In addition, there existed a strong geographical component to the distribution of alleles in group A, with only 4 such indel-based alleles found in the 11 accessions collected north of Lake Tiberias, mostly along the border with Lebanon. All four alleles were also found in accessions collected farther south, suggesting that the low level of diversity in northern germplasm may be a result of limited dispersal of alleles from the south rather than divergence of northern and southern lineages.

### Analysis of all Pisum SGR sequences

When 63 additional alleles from *P. sativum* were added to the alignment and a Bayesian phylogenetic analysis performed, the *P*. *fulvum* sequences continued to group primarily into two clusters, and these clusters fell into different regions of the tree for the genus (**Figure 2** and **Supplemental Table S2**). **Figure 2** presents only the tree developed from the manual alignment of the sequences, but very similar trees were produced from the MUSCLE and MAFFT alignments (data not shown). Rooting of the tree with a *P. fulvum* accession or the sequence from *Lens culinaris* rearranged the branching pattern but consistently placed the two *P. fulvum* groups in different regions of the tree, with *P. sativum* sequences on intermediate branches.

The exceptional sequence (PI 595941) that had been isolated on its own branch outside of groups A and B in the phylogeny generated from primarily *P. fulvum* sequences (**Figure 1**), clustered closely with sequences obtained from *P. sativum* landraces from Afghanistan and Pakistan in **Figure 2**. No other *P. fulvum* SGR allele possessed a structure closely resembling those of the three *P. sativum* landraces clustering with PI 595941

Positions of several of the lower branches of the genus tree changed depending on what SGR sequence was used to root the tree, and a number of the ultimate branches ended in polytomies, but most of the branches separating the principal groups of *P. sativum* accessions from each other and from the two *P*. *fulvum* groups displayed excellent support. We examined the alignment in **Supplemental Table S2** for sequence fragments that could be used to distinguish the two species. However, although there were numerous instances where a section of the aligned sequences was present primarily in *P. sativum*, or *P. fulvum* group A or group B accessions, we did not observe any case where a section of the sequence was (1) limited to one group or taxon and (2) present in all members of that taxon. Perhaps equally interesting was the observation that in many instances sections of sequence present in some or all of the *P. sativum* sequences were matched by sequences in only one of the *P. fulvum* groups. For instance, the sequence 200 to 207 (**Supplemental Table S2**) was present in most *P. sativum* but found only in group B of *P. fulvum*. The same was true for sequences at positions 410 to 424, 574 to 600, and 1863 to 1920. In contrast, only a limited number of cases existed of sequences common to most *P. sativum* and at least some *P. fulvum* group A accessions but lacking in group B accessions, the section between positions 1244 and 1324 (**Supplemental Table S2**) being the only example of any length. Hence, based on indel arrangement, the *P. sativum* accessions appeared to represent a group intermediate between the two *P. fulvum* groups with group B displaying greater diversity than group A and greater overlap with *P. sativum*.

## Discussion

The third intron of the *SGR* gene was found to be highly polymorphic in the *Pisum fulvum* accessions analyzed, with 57 alleles present in 82 non-synonymous accessions. This high level of polymorphism allowed the unique identification of most of the *P. fulvum* accessions in the USDA germplasm collection while verifying the synonymy of several pairs of accessions obtained from different sources. In combination with previous results (Weeden et al., 2021) these findings establish that the entire genus exhibits hypervariability within the intron. In contrast to intronic regions previously examined in *Pisum* (Jing et al., 2007; Weeden, 2018), the SGR intron displayed nearly as much indel polymorphism as single nucleotide variation, greatly simplifying allele identification. Many alleles could be distinguished simply by size on agarose gels, and restriction fragment analysis could be used to assign alleles to Group A or Group B when sequences were not available. The impressive polymorphism observed for the intron appeared to be produced by the complex repeat structure of the amplified fragment. Multiple repeats in a head-to-tail arrangement and 60 to 100 bases long formed the fundamental structure of the intron, and the number of these repeats, changes in repeat structure, and the presence of numerous indels and tandem duplications, were responsible for much of the polymorphism observed.

Given the hypervariability found in this intron, we evaluated whether the variation in nucleotide substitutions and indels detected within this intron could be due mostly to convergent evolution (i.e., a form of homoplasy) or to homology (similarity due to common ancestry). We assessed homoplasy versus homology using different DNA sequence alignments. One alignment was generated manually (as described in the Material and Methods section). The other alignment was generated using multiple refinements in MUSCLE with no subsequent manual refinement. The manual alignment resulted in an aligned SGR intron sequence length of 7932 nucleotide sites, which included 565 parsimony informative sites (nucleotide sites where at least two nucleotides were shared by at least two alleles). The alignment by MUSCLE resulted in an aligned sequence length of 3437 nucleotide sites, which included 955 parsimony informative sites (more site variation because deletions were shorter and insertions longer and more overlapping). With either alignment, relatively little homoplasy was detected, as measured by a parsimony analysis in PAUP, where the retention index (ri) derived from the manual alignment was ri=0.9026 and that derived from the MUSCLE alignment ri=0.8114. An ri close to 1.0 signifies all character evolution is happening at internal nodes and nucleotide site similarity is mostly homologous (minimum homoplasy). An ri close to 0 indicates character evolution is occurring mostly along terminal branches and nucleotide site similarity is due mostly to other than homology, such as convergent gain (i.e., maximum homoplasy). Those observed ri values for either alignment suggest most SGR site evolution is occurring along internal branches and thus has the power to group SGR alleles into clades. This finding of low levels of homoplasy among alternative SGR intron alignments is bolstered by a BEAST analysis for rates of substitution where the *Pisum* SGR crown clade was fixed at 5 Ma (biased young). For the manual SGR intron alignment, the estimated rate of substitution was 1.9 x 10^-8^ substitutions/site/year and for the MUSCLE alignment 6.3 x 10^-8^ substitutions/site/year. These values are perhaps an order of magnitude faster than expected average rates of substitution at nuclear loci, but they are much lower than those calculated for microsatellites in *Pisum* [about 5 x 10^-3^ substitutions/locus/year, (Cieslarová et al., 2011)] and are not so excessively fast to invoke concerns of high levels of homoplasy. Low levels of homoplasy are also suggested ultimately by preliminary parsimony and Bayesian analyses that resulted in many SGR intron branches being associated with high branch support values (e.g., posterior probabilities of 0.95-1.00). A relevant summary point here is that no conflict was detected among the analyses of the different alignments with respect to clades resolved with high branch supports (i.e., posterior probabilities of 0.95-1.00) especially in regard to resolved clades related to the questions being addressed in this study.

The *SGR* genotype proved much better for distinguishing *P. fulvum* accessions than morphological traits not just because the former was considerably more variable, but also because much of the morphological variation displayed incomplete penetrance or was influenced by environmental factors. For the eight cases of synonymous pairs of accession, the identity of each pair was verified by *SGR* allele, but most exhibited slightly different phenotypes for some of the traits examined. Preliminary genetic studies on three small F_2_ populations indicated that most of the morphological variation possessed a heritable component, in that crosses between accessions with the same morphotype did not exhibit segregation in the F_2_, whereas crosses between accessions differing in one or more traits produced F_2_ progeny that segregated for the respective traits (N. Weeden and K. McPhee, unpublished). However, the genetic basis for the traits may be more complex than that described for *Pisum sativum* (Blixt, 1972)

The evolution of sequences with a repeat structure is known to be facilitated by polymerase slippage or imperfect pairing of homologous regions during replication (Eckert and Hile, 2009). Some of the repeats in the intron could have arisen in this fashion. Certainly, the GAG insertion within a GAGAG repeat in accessions Pf 25 and Pf 31 is a candidate for such a mechanism. Some longer (20-60 nt) repeats that are present as 2 to 5 tandem copies in certain accessions are also potential candidates. However, the ‘major repeat’ structure observed in the central portion of most sequences is harder to explain. If the change in number of the repeats was produced by this mechanism most of the changes must have been generated early in the evolution of the species because the individual units appear highly eroded, and the group A and group B structures differ markedly. Even the overall repeat structure observed among accessions can vary considerably. That of JI 2205 is somewhat regular, with almost seven recognizable repeat units that extend into the 3’ conserved region (**Supplemental Figures S1, S2**), whereas in Pf 10 the major repeat structure is barely recognizable because half way through the sequence (starting with that section containing the perfect GTAAGT motif) the major repeat changes to a longer form containing two GA-rich sections.

Another mechanism for generation of repetitive regions of a genome is through repeated insertions or movement of retrotransposons. The *Pisum* genome consists to a large extent of such dormant or residual retrotransposon sequences (Murray et al., 1981; Macas et al., 2015); however, the major repeat units found in intron 3 of *SGR* are not found elsewhere in the pea genome, nor are they similar to transposable elements known from other genomes. Thus, the repeats in SGR do not appear to be transposable elements or relics from retrotransposon activity. At present, the underlying mechanism resulting in the rapid evolution of this intron eludes us. The sequence appears to have risen *de novo* within the Fabeae tribe of the Fabaceae, being found in *Pisum* but not *Lens, Cicer*, or *Medicago* (Weeden et al., 2021). A more extensive investigation of its distribution within the Fabeae is underway.

The division of *P. fulvum SGR* sequences into two major groups, initially observed in Weeden et al. (2021), was verified in a much larger sample of the species. This inclusion of additional *P. fulvum* alleles in the Baysian analysis did not alter the groupings of the *P. sativum* clades reported in Weeden et al. (2021) except for the *P. sativum* group A, which was split because one of its members (W6-26109) was used to root the tree presented in the current manuscript. Similarly, the positions of the two *P. fulvum* clades relative to the *P. sativum* groups were basically the same in both analyses. Despite the very large number of alleles identified in *P. fulvum*, the separation of these into two groups was well-supported, with only the sequence of PI 595941 falling outside the two clusters.

The high similarity between the allele in PI 595941 and *P. sativum* landraces from Afghanistan and Pakistan strongly suggests that this accession has an ancestor of these landraces in its pedigree. The landraces belong to a relatively distinct lineage of domesticated pea occasionally grown from Iran to Pakistan (Weeden and Wolko, 1988). This lineage, appears to have formed relatively soon after initial domestication events had fixed indehiscent pods, smooth testa, and non-dormant seeds in the domesticated pea lineage, probably within the last 20,000 years. It is not readily apparent how such a hybridization and subsequent backcrossing to *P. fulvum* germplasm would have occurred given the current lack of sympatry of the two taxa. PI 595941 is also known as JI 1796, which was reported to be an outlier in terms of its slightly higher level of heterozygosity in the study by Smýkal et al. (2017). Our sample of PI 595941 has been taken through several generations of selfing in the USDA germplasm system and should be relatively homozygous. Its morphological phenotype reflects that of a typical *P. fulvum* accession. Jing et al. (2007) did not note anything peculiar about JI 1796. Thus, at present there is little additional support for a possible hybrid origin of this accession. However, it may be appropriate to treat PI 595941 as an atypical *P. fulvum*.

Overall, the level of *SGR* polymorphism in *P. fulvum* was slightly less than that found in *P*. *sativum* subsp. *elatius* (Weeden et al., 2021). However, the reduction was not as great as reported by Hellwig et al. (2020b) in their more extensive comparison of genetic diversity in the two species. In particular, the extraordinary diversity (85% unique alleles) that we observed in group B *SGR* sequences was greater than what we found in our previous study of *P. sativum* subsp. *elatius*. Our sampling procedures for both the *P. sativum* subsp. *elatius* and that for *P*. *fulvum* were similar, selecting most accessions available but not including all samples from what appeared to be clustered collections. For instance, we selected only one accession between Pf 50 and Pf 59 because all of these were collected in the area just west of Jerusalem and all clustered in one region of the phylogenetic tree of Hellwig et al. (2020b). We did not target accessions based on whether we thought they might belong to an A and B grouping (a characterization we were unable to predict at the time of sampling).

Based on this selection criteria, we feel the differences observed between group A and group B in range and sequence diversity are real and reflect evolutionary processes. The distribution of group B accessions suggests they are geographically restricted to a more arid environment or perhaps represent relictual populations that have not been able to spread to the north. If the latter is the case, perhaps their more southern distribution protected them from population bottlenecks during the last ice age, the reason Hellwig et al. (2020b) suggested for the greater reduction in genetic diversity they observed in *P. fulvum* relative to *P. sativum* subsp. *elatius*. This possibility was supported by the finding of most of the sequence diversity in group A accessions was also found in southern populations. Alternatively, the rapid rate of evolution for the SGR intron clearly generated more polymorphism at this locus than at those sequences studied by Hellwig and co-workers. The rapid evolution of the SGR sequence may have been sufficient to obscure the effects of an ice age population bottleneck.

As many of the *P. fulvum* accessions we examined were also analyzed in the studies by Naim-Feil et al. (2017) and Hellwig et al. (2020b), a comparison of the phylogenetic results seemed appropriate. Our analysis contained 41 accessions also included in the study by Hellwig et al. (2020b). Most of the branches of the tree presented in Hellwig et al. (2020b) had poor support, so we limited our comparison to their first major dichotomy (to check for a group A/group B division) and the several terminal branches for which the authors reported high clade support values. When we totaled the number of group A and group B accessions found in subset of accessions formed by the first dichotomy of the tree in Figure 2 of Hellwig et al. (2020b) we determined that there were 10 subgroup A accessions and 7 subgroup B accessions in the upper branch and 13 subgroup A and 11 subgroup B accessions in the lower branch, strongly indicating that this tree did not separate *P. fulvum* accessions into our A and B groups. The failure of Hellwig et al. (2020b) to find evidence of an A and B clade in *P. fulvum* suggests that our study is detecting SGR allele evolution but not necessarily organismal evolution in *Pisum fulvum*.

Hellwig et al. (2020b) found a close relationship between Pf 4 and Pf 21, which in our study both had the 607a allele in subgroup A-2. Another member of this subgroup in our study, Pf 67, was placed near the Pf 4/Pf 21 branch in Hellwig’s phylogeny, but the support value was not above 80%. The authors noted that three accessions Pf 10, Pf 11 and Pf 13 showed a close relationship, whereas in our study only Pf 11 and Pf 13 clustered in subgroup B-8 (with different SGR alleles), with Pf 10 possessing a highly divergent subgroup B-5 allele. The only other comparison available was for accessions along the Lebanese border. Five accessions coming from this region (Pf 19, Pf 25, Pf 26, Pf 31, and Pf 32) clustered near each other on the phylogeny of Hellwig et al. (2020b) (although UPGMA bootstrap support was below 80%) and belonged to our subgroup A-1-b. However, another accession in subgroup A-1-b, Pf 28, as well as an accession we did not test but would predict that it should exhibit an A-1-b SGR allele on the basis of its collection site, clustered together in the Hellwig phylogeny with strong support but in a different region of the tree from the aforementioned five accessions. Thus, even for groups predicted by SGR sequence to be closely related, other DNA-based analyses only partially confirm such groupings.

Fewer accessions (34) were found to overlap between our study and that of Naim-Feil et al. (2017), but using the same approach for analyzing whether an A/B grouping existed in their analysis, we found 6 group A and 9 group B accessions in the subset of accession on the left branch of the first dichotomy in their Figure 2 and 10 group A and 9 group B in the right side. Again, there appears to be little evidence for an A/B grouping of *P. fulvum* accessions in this analysis of 91 loci scattered across the genome. The only well-supported clades found by Naim-Feil et al. (2017) based on SNP analysis involved pairs of accessions for which we did not have data on both members of the pair.

The analysis of variation of a single locus always is associated with the risk that evolution at that locus does not reflect the more general evolution of the taxon (Doyle, 1992). We suspect that sequence evolution observed for the third intron of the *STAYGREEN* locus in *P. fulvum* is a case in point. The division of this species into two lineages based on *SGR* genotype was not supported by two previous studies. The two previous studies differed from the current investigation in their use of many markers scattered across the genome. An explanation for the conflicting results is that the region of the genome containing the SGR locus has at some time experienced strong selection pressure, resulting in the formation of the two SGR lineages. Selection may be ongoing but likely started near the time *P. fulvum* began diverging from *P. sativum* because each of the lineages share sequence fragments with *P. sativum* that they do not share with each other. An alternative hypothesis for the inconsistent results is that the SGR fragment is experiencing an exceptionally high rate of change through a currently unknown mechanism, possibly making it inappropriate for comparative phylogenetic studies. The two hypotheses can be tested by examining variation at loci closely linked to SGR to determine if they also divide the species into groups A and B. If not, the selection hypothesis can be rejected.

Genetic drift could also be a factor involved in forming the observed distribution of SGR genotypes if one assumes that the A and B types were widespread and sympatric before the last ice age and many isolated populations of *P. fulvum* exist. Hellwig et al. (2020b) postulated that the effective population size of *P. fulvum* was much larger before the last ice age, suggesting that the first criterion may be fulfilled. Relatively low outcrossing rates (supported by the self-compatible nature of *Pisum fulvum*, but called into question by the results of Hellwig et al., 2021b) could satisfy the second, thus explaining the mosaic of group A and group B populations currently observed in the region south of Lake Tiberias.

Irrespective of the cause of the variation in the third intron of SGR, such variation appears to be an excellent tool for fingerprinting *Pisum fulvum* accessions. Except for leaflet serration pattern and minor variations of pigment pattern on stems, pods and seeds, the species portrays a relatively consistent morphology, making identification of accessions using morphology extremely difficult. The recent collection of an extensive sample of *P. fulvum* germplasm from Israel (Naim-Feil et al., 2017), as well as the availability of additional accessions from Turkey, Jordan, and Syria in various germplasm repositories (Aydin et al., 2021; Rispail et al., 2023) makes this species an excellent candidate for the study of evolution and adaption in a close relative to an important crop. Although Bayesian or PAUP analysis of SGR sequences does not appear to reflect the overall evolutionary history of the species, correlations between SGR genotype and geographical or ecological parameters can provide insight into several aspects of its biology and phylogeny. A better understanding of the molecular mechanisms generating the sequence variation may allow a more critical evaluation of the observed changes in indel structure and help clarify phylogenetic relationships.

## Data availability statement

The datasets presented in this study can be found in online repositories. The names of the repository/repositories and accession number(s) can be found in the article/**Supplementary Material.**

## Author contributions

NW performed most of the handling of plants and laboratory work, some data analysis and writing of the manuscript. ML provided the phylogenetic analysis, some of the experimental design and writing. SA supplied much of the germplasm, background information on accessions and helped with the writing. CC supplied germplasm and assisted with the writing. KM provided laboratory support and help with the writing. All authors contributed to the article and approved the submitted version.
