# Supplementary figures and images for: A hypervariable intron of the STAYGREEN locus provides excellent discrimination among Pisum fulvum accessions and reveals evidence for a relatively recent hybridization event with Pisum sativum
Source: Front Plant Sci. 2023 Aug 25;14:1233280. doi: 10.3389/fpls.2023.1233280 (PMC10492584; doi:10.3389/fpls.2023.1233280)

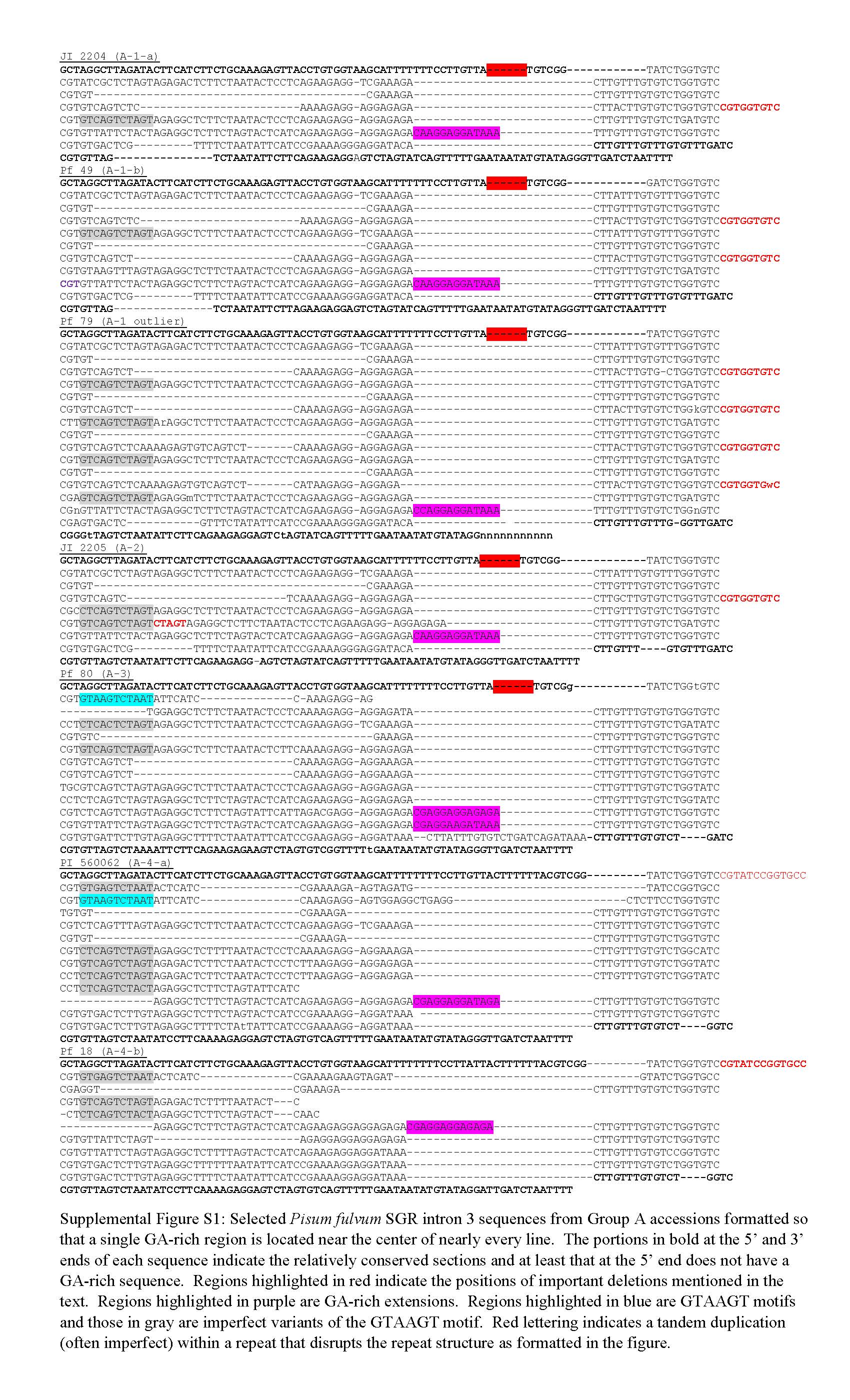

Supplement: Supplementary file 1 [file Image_1.jpeg]

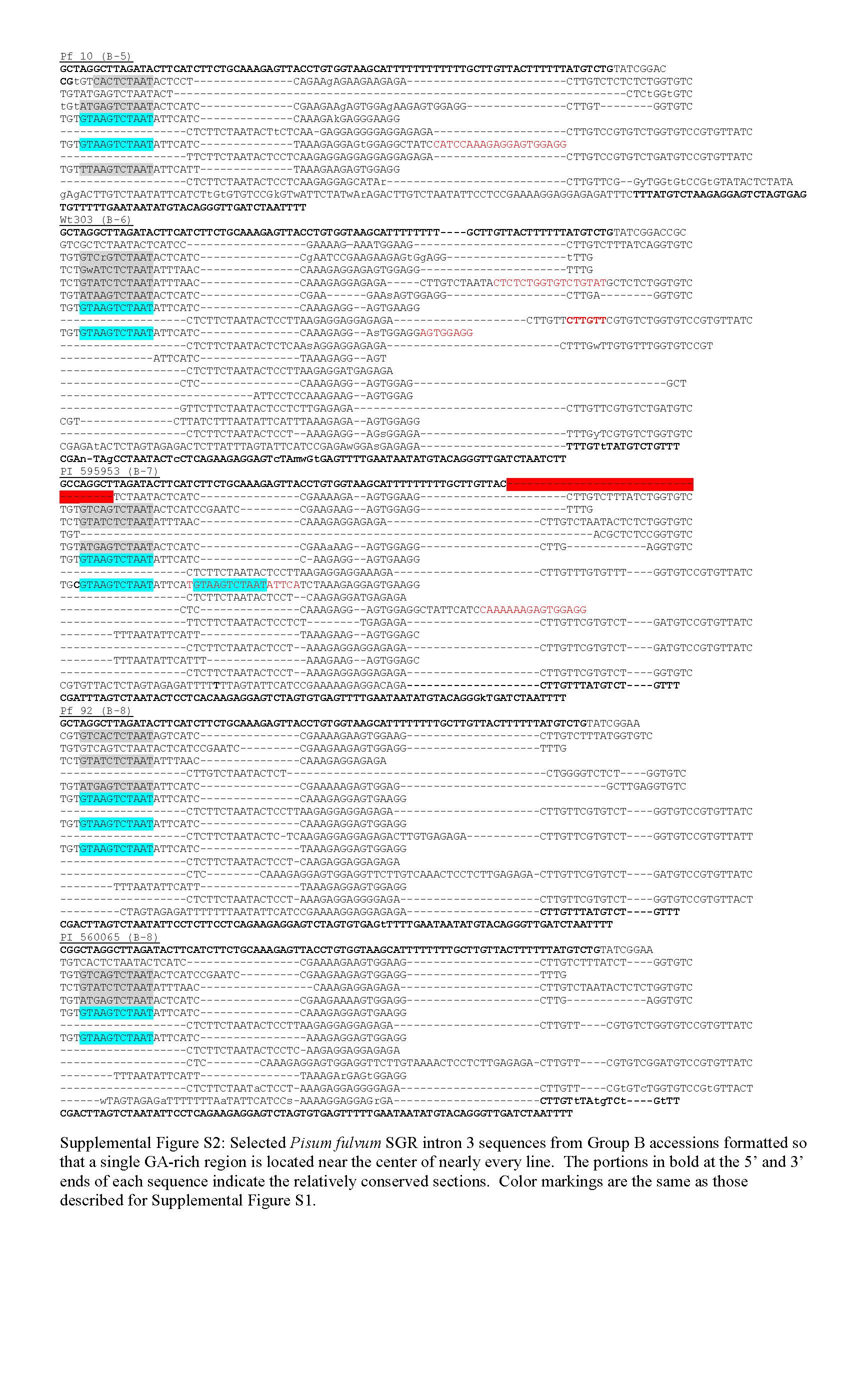

Supplement: Supplementary file 2 [file Image_2.jpeg]
