# Supplementary material for: A hypervariable intron of the STAYGREEN locus provides excellent discrimination among Pisum fulvum accessions and reveals evidence for a relatively recent hybridization event with Pisum sativum
Source: Front Plant Sci. 2023 Aug 25;14:1233280. doi: 10.3389/fpls.2023.1233280 (PMC10492584; doi:10.3389/fpls.2023.1233280)
Supplement: Supplementary file 3 [file Table_1.docx]

| Supplemental Table S1. The 90 accessions of *Pisum fulvum* examined, their respective SGR allele, collection location, and phenotypes for six morphological characters | | | | | | | | | |
| --- | --- | --- | --- | --- | --- | --- | --- | --- | --- |
| Accession designation^1^ | Synonyms | SGR allele^2^ | Collection coodinates | Testa color | Testa anthocyanins | Testa mottle | Leaf axil pigmentation | Serrate leaflets | Anthocyanin dots on pod |
| Group A |  |  |  |  |  |  |  |  |  |
| A-1-a |  |  |  |  |  |  |  |  |  |
| PI 595932 | PIG 112 | 536a | no data | olive | minor streaks | yes | none | strong | purple haze |
| PI 595947 | VIR2523 | 536a | no data | olive | minor streaks | yes | none | strong | purple haze |
| VIR2523 | PI 595947 | 536a | no data | olive | minor streaks | yes | none | strong | purple haze |
| PI 595948 | VIR3397 | 536a | no data | olive | minor streaks | yes | none | strong | purple haze |
| VIR3397 | PI 595948 | 536a | no data | olive | minor streaks | yes | none | strong | purple haze |
| JI2204 | SA 1607 | 536a | no data | olive | faint dots | yes | none | dentate | purple haze |
| Pf24 | W6-57828 | 536a | 33.09, 35.48 | brown | dots | yes | faint dots | entire | faint |
| Pf29 | W6-57833 | 536a | 32.99, 35.42 | dark | unknown | unknown | none | weak | yes |
| Pf65 | W6-57867 | 536a | 33.08, 35.83 | dark | unknown | unknown | no data | no data | no data |
| Pf70 | W6-57872 | 536a | 31.92, 35.02 | brown | none | no | none | entire | no data |
| Pf71 | W6-57873 | 536b | 32.49, 35.06 | light brown | none | no | faint dots | weak | no data |
| Pf72 | W6-57874 | 536b | 31.55, 34.86 | light brown | none | no | strong dots | weak | no |
| Pf132 | W6-57804 | 536b | 31.87, 35.13 | dark | unknown | unknown | no data | entire | faint |
| Pf57 |  | 536c | ~31.7, 35.0 | brown | none | yes | none | entire | no |
| Pf136 | W6-57808 | 548 | 32.26, 34.84 | dark | unknown | unknown | none | weak | faint |
| A-1-b |  |  |  |  |  |  |  |  |  |
| Pf49 | W6-57853 | 693 | 31.68, 34.92 | dark | unknown | unknown | dots | entire | strong flecking |
| Pf63 | W6-57865 | 694a | 33.27, 35.74 | brown | intense dots | no? | none | weak | no |
| Pf116 | W6-57789 | 694a | 32.00, 35.05 | dark | unknown | unknown | dots | entire | yes |
| Pf120 | W6-57793 | 694a | 32.08, 35.09 | dark | unknown | unknown | dots | weak | yes |
| Pf19 | W6-57821 | 694b | 33.27, 35.57 | dark brown | unknown | unknown | intense dots | strong | yes |
| Pf26 | W6-57830 | 694c | 33.04, 35.41 | dark | unknown | unknown | dots | weak | yes |
| Pf28 | W6-57832 | 694d | 33.03, 35.41 | tan | none | no | dots | strong | faint |
| Pf32 | W6-57837 | 694e | 33.07, 35.27 | dark brown | unknown | no | none | entire | strong flecking |
| Pf69 | W6-57871 | 694f | 33.04, 35.19 | dark | intense dots | no? | none | entire | faint |
| Pf85 | W6-57882 | 694g | 32.10, 35.40 | olive | none | no | none | weak | yes |
| PI 595951 | WL2143 | 694g | no data | dark brown | unknown | no? | dots | entire | yes |
| Pf25 | W6-57829 | 697a | 33.07, 35.45 | dark brown | unknown | unknown | dots | entire | no |
| Pf31 | W6-57836 | 697b | 33.08, 35.29 | brown | none | unknown | none | entire | yes |
| A-1 outlier |  |  |  |  |  |  |  |  |  |
| Pf79 | W6-57879 | 1018 | 32.87, 35.51 | light brown | dots | no | none | entire | no data |
|  |  |  |  |  |  |  |  |  |  |
|  |  |  |  |  |  |  |  |  |  |
| A-2 |  |  |  |  |  |  |  |  |  |
| Pf4 |  | 607a | 32.48, 35.13 | olive to brown | none | no | intense ring | entire | no |
| VIR6070 | JI2205 | 607a | ~31.7, 35.2 | tan or dark | none or unknown | no | none | entire | faint |
| JI2205 | VIR6070 | 607a | ~31.7, 35.2 | olive to brown | none | yes | none | entire | yes |
| PI 560064 | L95, 703 | 607a | ~31.7, 35.0 | dark brown | none | no | dots | weak | yes |
| L95 | PI 560064, 703 | 607a | ~31.7, 35.0 | tan or brown | none | no | none | entire | yes |
| Pf67 |  | 607a | 31.74, 35.03 | brown | none | no | dots | entire | faint |
| Pf21 | W6-57826 | 607a | 33.17, 35.55 | dark | unknown | no | none | entire | yes |
| PI 433560 | W6-15045 | 607b | Israel | dark | unknown | unknown | dots | entire | no |
| W6-15045 | PI 433560 | 607b | Israel | brown | unknown | unknown | none | strong | purple haze |
| PI 595937 | JI1006, WBH 2142 | 607b | Israel | dark brown | none | no | dots | entire | no |
| A-2 outlier |  |  |  |  |  |  |  |  |  |
| Pf60 | none | 614 | ~31.9, 35.0 | dark | unknown | unknown | none | strong | no |
| A-3 |  |  |  |  |  |  |  |  |  |
| PI 531199 | CPI 134464 | 681 | 32.93, 35.51 | dark brown | none | no? | none | weak | no |
| Pf80 |  | 918 | ~32.0, 35.2 | brown | none | yes | none | weak | no |
| PI 595939 | JI1011 | 918 | 32.36, 34.88 | dark brown | none | unknown | none | weak | yes |
| PI 595940 | JI1012, NGB102145 | 918 | Israel | dark brown | none | unknown | none | weak | yes |
| Pf43 |  | ~1066 | 31.66, 35.11 | brown | dots | yes | faint dots | weak | no |
| A-3 outlier |  |  |  |  |  |  |  |  |  |
| PI 595935 | IC63466 | 864 | Syria | dark | unknown | unknown | none | strong | strong flecking |
| A-4-a |  |  |  |  |  |  |  |  |  |
| Pf87 |  | 846 | 32.04, 35.35 | brown | none | no | none | weak | faint |
| Pf42 |  | 877 | 31.60, 35.18 | dark brown | unknown | unknown | dots | entire | no |
| PI 560062 | L93, 701,  CPI 134466 | 948 | 31.77, 35.21 | dark brown | unknown | unknown | dots | weak | yes |
| Pf112 |  | 955 | 31.44, 35.02 | dark | unknown | unknown | dots | entire | yes |
| Pf105 |  | 1018 | 32.74, 35.04 | light brown | intense dots | no? | dots | dentate | no |
| A-4-b |  |  |  |  |  |  |  |  |  |
| Pf18 |  | 658 | 32.74, 35.02 | light brown | intense dots | no? | dots | entire | no |
| Pf138 |  | 785 | 32.75, 35.01 | light brown | intense dots | no? | dots | entire | yes |
| Group B |  |  |  |  |  |  |  |  |  |
| B-5 |  |  |  |  |  |  |  |  |  |
| Pf10 |  | 705 | 31.35, 35.04 | brown | intense dots | no? | none | strong | strong flecking |
| Pf74 |  | >1000 | ~32.6, 35.75 | dark | unknown | unknown | dots | entire | faint |
| B-6 |  |  |  |  |  |  |  |  |  |
| VIR 6071 | PI 595943 | 915 | no data | dark brown | unknown | unknown | dots | strong | yes |
| PI 595943 | JI2206, VIR6071 | 915 | no data | dark | unknown | unknown | dots | strong | yes |
| Wt303 | W6-15046 | 933 | no data | olive | intense dots | no? | none | entire | yes |
| PI 595950 | WL2140 | 933? | Israel | brown | intense dots | no? | dots | entire | yes |
| B-7 |  |  |  |  |  |  |  |  |  |
| Pf84 |  | 594 | 32.29, 35.44 | olive-tan | none | no | dots | entire | faint |
| Pf111 |  | 617 | 32.55, 34.95 | dark | solid cover | unknown | dots | entire | yes |
| PI 595949 | NGB102147 | 722a | no data | olive | intense dots | no? | dots | weak | purple haze |
| PI 560066 | L97, 707 | 722a | ~33.1, 35.2 | dark brown | unknown | no? | dots | weak | faint |
| L97 | CPI 134470 | 722a | ~33.1, 35.2 | brown | none | no | dots | entire | yes |
| Pf5 |  | 722a | 32.24, 35.04 | brown | none | no | none | weak | faint |
| Pf48 |  | 722a | 31.71, 34.91 | dark | unknown | unknown | dots | entire | no |
| PI 560061 | L91, JI224, WBH2029 | 722b | 31.46, 35.1 | tan | none | no | dots | entire | yes |
| PI 595953 | JI1392, NGB101256 | 775 | no data | dark brown | unknown | unknown | dots | strong | yes |
| PI 560063 | L94, 702 | 864 | ~33.0, 35.5 | brown | intense dots | no? | dots | entire | no |
| L94 | 702, CPI 134467 | 864 | ~33.0, 35.5 | tan | intense dots | no? | none | entire | no |
| Pf9 | W6 57887 | 869 | 31.80, 34.93 | brown | none | yes | dots | entire | yes |
| Pf73 |  | 869 | ~32.85, 35.5 | dark | unknown | unknown | dots | entire | yes |
| PI 595946 | Line 1256, PIG296 | 871 | Turkey | brown | intense dots | no? | dots | entire | faint |
| PI 595938 | JI1010, NGB102144 | 874 | 31.36, 34.54 | dark | unknown | unknown | dots | strong | no |
| PI 595933 | ATC 113 | 906 | no data | olive | intense dots | no? | dots | entire | strong flecking |
| PI 595934 | ATC 114 | 906 | no data | light brown | intense dots | no? | intense ring | weak | yes |
| B-8 |  |  |  |  |  |  |  |  |  |
| Pf66 |  | 529 | 32.66, 35.23 | brown | none | no | faint dots | dentate | yes |
| Pf95 |  | 695 | 32.64, 35.54 | light brown | none | no | intense dots | strong | few |
| Pf11 |  | 700 | 31.35, 35.06 | light brown | none | no | dots | dentate | no |
| Pf46 |  | 713 | 31.93, 34.84 | dark | unknown | unknown | dots | entire | faint |
| PI 560065 | L96, 706 | 756 | ~31.3, 34.4 | dark brown | unknown | unknown | dots | dentate | no |
| Pf17 |  | 839 | 32.14, 34.97 | dark | unknown | unknown | dots | entire | yes |
| Pf13 |  | 841 | 31.46, 34.91 | light brown | faint dots | yes | dots | strong | strong flecking |
| Pf92 |  | 873 | 32.55, 35.34 | dark | unknown | unknown | dots | weak | yes |
| PI 560067 | L98, 708 | 912 | ~31.7, 35.2 | dark brown | unknown | no | none | entire | no |
| Pf35 |  | 997 | 33.08, 35.20 | brown | none | no | intense dots | weak | no |
| Pf40 |  | >1100 | 31.65, 35.19 | olive | dots | yes | none | weak | no |
| B-8 outliers |  |  |  |  |  |  |  |  |  |
| Pf94 |  | 823 | 32.64, 35.52 | dark brown | unknown | unknown | none | entire | no |
| Pf101 |  | ~966 | 32.00, 34.96 | dark | unknown | unknown | intense dots | weak | yes |
| Hybrid-derived |  |  |  |  |  |  |  |  |  |
| PI 595941 | JI1796 | 894 | 31.46, 35.1 | dark | unknown | unknown | dots | dentate | yes |

^1^Accessions highlighted in red are supposedly synonymous with the accession immediately above them in the table.

^2^Number indicates length in nucleotides, letter (when present) indicates sequence variant.
