# Supplementary material for: A hypervariable intron of the STAYGREEN locus provides excellent discrimination among Pisum fulvum accessions and reveals evidence for a relatively recent hybridization event with Pisum sativum
Source: Front Plant Sci. 2023 Aug 25;14:1233280. doi: 10.3389/fpls.2023.1233280 (PMC10492584; doi:10.3389/fpls.2023.1233280)
Supplement: Supplementary file 5 [file Table_3.docx]

**Supplemental Table S3. Positions in Supplemental Table S2 of sequences distinguishing *P. fulvum* groups A and B**

| Unique to nearly all group A accessions | Unique to nearly all group B accessions |
| --- | --- |
| 1182-1211 | 247-270 |
| 1242-1248 | 369-424 |
| 1289-1324 | 511-532 |
| 1645-1684 | 540-600 |
| 4945-5007 | 655-681 |
| 5659-5742 | 1838-1920 |
|  | 3914-4052 |
|  | 4070-4105 |
|  | 5827-5851 |
|  | 5892-5910 |
|  | 7402-7442 |
